# A Rare Case of Uterine Embryonal Rhabdomyosarcoma in a Postmenopausal Woman

**DOI:** 10.7759/cureus.104672

**Published:** 2026-03-04

**Authors:** Satvika Nimmagadda, Nicole Long

**Affiliations:** 1 Internal Medicine, Edward Via College of Osteopathic Medicine, Blacksburg, USA; 2 Obstetrics and Gynecology, Seasons for Women, Holston Medical Group, Abingdon, USA

**Keywords:** elderly, embryonal rhabdomyosarcoma, palliative care, postmenopausal bleeding, soft tissue sarcoma, uterine neoplasms

## Abstract

This report describes a rare presentation of embryonal rhabdomyosarcoma (ERMS) in a 76-year-old postmenopausal woman who presented with several months of unreported vaginal bleeding followed by obstructive urinary symptoms. Imaging revealed a large uterine mass, and biopsy with immunohistochemical staining confirmed ERMS characterized by high-grade histopathologic features. Given her advanced age, comorbidities, poor functional status, and extensive tumor burden, the patient opted for palliative care focused on comfort and quality of life. This case highlights the diagnostic challenges, aggressive nature, and poor prognosis associated with adult ERMS. It reinforces the importance of promptly evaluating postmenopausal bleeding and raising clinician awareness to facilitate earlier diagnosis and improve outcomes in elderly patients.

## Introduction

Rhabdomyosarcoma (RMS) is the most frequently diagnosed soft tissue sarcoma in the pediatric population, predominantly affecting those under six years of age [1]. It originates from primitive mesenchymal cells, which typically develop into skeletal muscle [2]. RMS in adults is rare, accounting for only 3% of soft-tissue sarcomas and less than 1% of all adult solid tumors [1]. The embryonal subtype accounts for the majority of cases and can develop at any site, most commonly in the genitourinary tract and the head and neck region [2]. Among adult females, the incidence is approximately 0.4-1%, with the vagina most commonly affected and the cervix or uterus far less frequently involved [3]. Very few cases of uterine embryonal rhabdomyosarcoma (ERMS) have been reported in postmenopausal women, making this presentation exceptionally uncommon. Moreover, diagnosis in adults is often delayed due to nonspecific symptoms, leading to more advanced disease at presentation [1].

Diagnosis is typically established with MRI or CT and confirmed by histopathology, which shows small, round, or spindle-shaped tumor cells and rhabdomyoblasts within a myxoid matrix [4]. Immunohistochemistry is usually positive for desmin, myogenin, and MyoD1 (myogenic differentiation 1), confirming rhabdomyoblastic differentiation [2]. Prognostic factors include tumor size, primary site, presence of metastases at diagnosis, and extent of surgical resection [5]. Adult uterine ERMS tends to be more aggressive, less responsive to therapy, and associated with poorer survival compared to pediatric cases [6]. Treatment is usually multimodal, combining surgery, chemotherapy, and radiation therapy [2]. However, no standardized guidelines exist for adults, and management is often extrapolated from pediatric regimens.

We present the case of a postmenopausal woman with abnormal uterine bleeding who was ultimately diagnosed with embryonal rhabdomyosarcoma of the uterus.

## Case presentation

A 76-year-old Caucasian woman with a BMI of 38.95 kg/m^2 ^and multiple comorbidities had intermittent postmenopausal bleeding beginning in January 2024 but did not seek care. Three months later, the bleeding had progressed to the passage of clots and was accompanied by diffuse abdominopelvic pain, decreased appetite, and nausea. Four months after symptoms started, in May 2024, she was evaluated by a gynecologist, and a dilation and curettage (D&C) was recommended; however, the procedure was pending cardiac clearance due to her comorbidities.

Before the D&C could be performed, she developed severe urinary retention and presented to the emergency department (ED) in July 2024. During workup for urinary retention, CT imaging revealed a markedly enlarged uterus (~13.5 cm sagittal diameter) with significant endometrial thickening and a heterogeneous mass suspicious for malignancy, compressing the urethra and bladder outlet. A Foley catheter was placed, and she was referred for an outpatient gynecologic evaluation. The day before this was scheduled, in August, she presented again to the ED with syncope and shortness of breath. She was diagnosed with bilateral pulmonary emboli and treated with thrombolytics, after which she was transitioned to long-term warfarin.

On examination at Gynaecology, she was afebrile and hemodynamically stable. Pelvic examination revealed a palpable mass with a distended bladder and foul-smelling, irregular tissue protruding from the vaginal canal just inferior to the urethral meatus. Speculum examination showed friable tissue along both vaginal sidewalls and the anterior wall. The cervix could not be visualized. Bimanual examination revealed bilateral parametrial involvement, an enlarged uterus, and replacement of the cervix with irregular, friable tissue. No active bleeding was observed. Examination was also notable for lower back tenderness without organomegaly. A prior transvaginal ultrasound was not available for review. 

Her gynecologic history included three pregnancies and two live births, a bilateral tubal ligation following her last pregnancy (year unknown), consistently negative Pap smears before discontinuation of cervical cancer screening at age 65, and menopause in her 50s. She denied any history of gynecologic malignancy or abnormal bleeding before 2024. Family history was notable for breast, cervical, and colon cancers, although the degree of relation was unknown. She denied tobacco, alcohol, or illicit drug use.

Laboratory studies at the ED demonstrated leukocytosis, normocytic anemia, hyponatremia, and hypoalbuminemia. Troponin was elevated in the setting of her pulmonary embolism. Inflammatory and coagulation markers were markedly abnormal, with elevated procalcitonin and a significantly increased international normalised ratio (INR). Urinalysis was positive for hematuria, nitrites, and leukocytes. A summary of these findings is presented in Table 1.

**Table 1:** Summary of abnormal laboratory findings INR: international normalized ratio

Laboratory Parameter	Initial ED Vist Values	Later ED Visit Values	Reference Range	Interpretation
White blood cell count	7.3	16	4.0–11.0 × 10³/µL	Leukocytosis
Hemoglobin	10.5	7.5	12–16 g/dL	Severe anemia
Hematocrit	29.8%	22.6%	36–46%	Decreased
Sodium	-	147	135–145 mmol/L	Mild hypernatremia
Albumin	2.4 g/dL	-	3.5–5.0 g/dL	Low
Troponin	289	-	<14 ng/L	HIgh
Procalcitonin	140	-	<0.5 ng/mL	Markedly elevated
INR	4.37	≥10.52	0.8–1.2	Significantly elevated
Urinalysis: RBC	-	30-50	0/HPF	Hematuria
Urinalysis: nitrites	-	2+	Negative	Positive
Urinalysis: leukocytes	-	500/µL	Negative	Pyuria

Initial endometrial biopsy yielded necrotic tissue. A repeat biopsy one month later demonstrated a poorly differentiated sarcomatoid malignancy with extensive necrosis. Immunohistochemistry revealed weak positivity for CKAE1/AE3 and p40, with focal positivity for desmin, myogenin, MyoD1, CK8/18, and CD10. The tumor was negative for SMA, CD45, S100, CD31, CD34, Melan-A, ER, and PR, excluding carcinoma, lymphoma, melanoma, endothelial tumors, and other soft tissue neoplasms from the differential diagnosis. 

**Figure 1:**
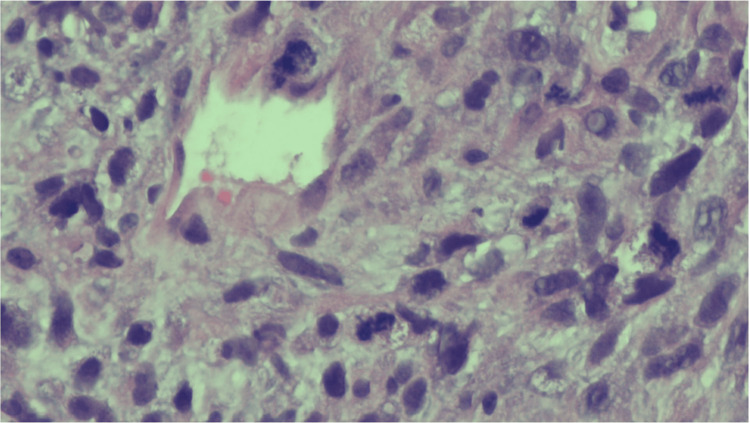
Microscopic image showing areas of multiple mitoses. Spindle tumor cells are present, with some exhibiting bizarre nuclei.

**Figure 2:**
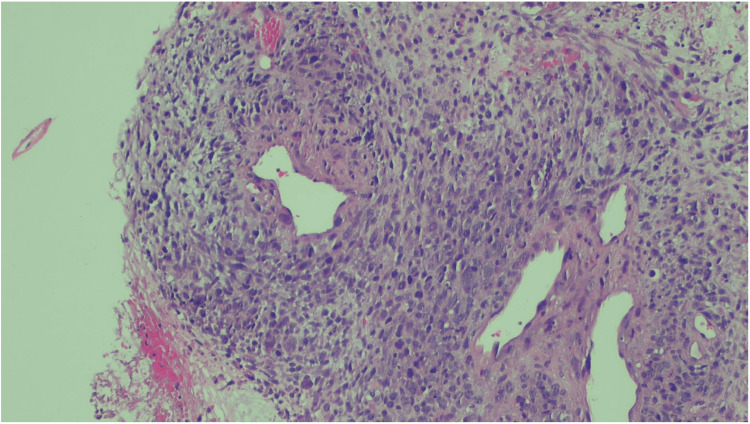
Microscopic image showing viable spindle tumor cells surrounding large vessels in a peritheliomatous pattern, with intervening myxoid and edematous zones.

**Figure 3:**
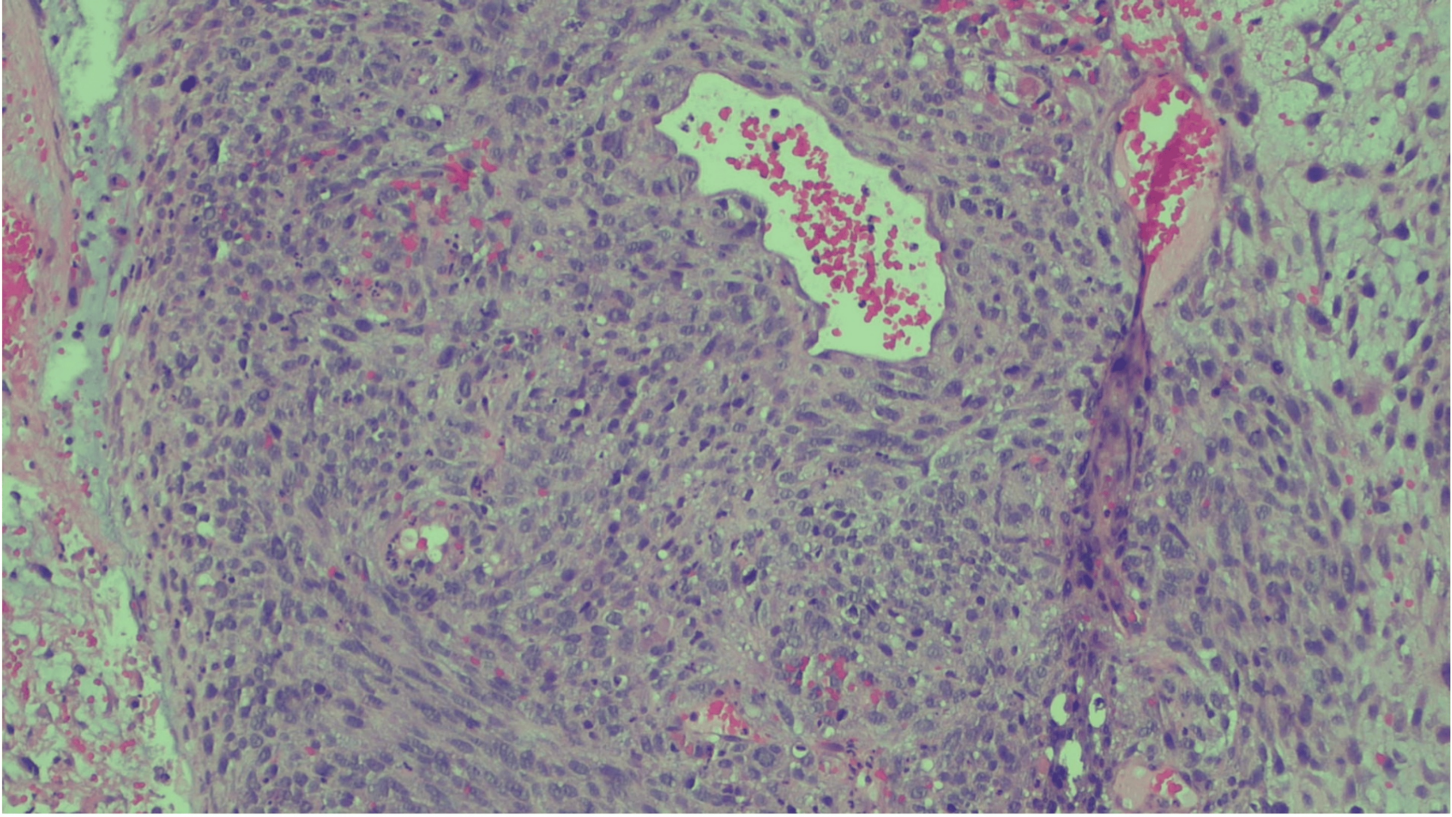
Additional microscopic field showing spindle tumor cells in a peritheliomatous pattern with myxoid changes from another region of the tumor.

Histology demonstrated spindled tumor cells with a high mitotic index and occasional “monster nuclei” (Figure 1). Additional sections showed perivascular whorling (peritheliomatous pattern) of tumor cells with intervening myxoid and edematous zones (Figures 2, 3). Other findings included hypercellularity, abundant necrotic debris, and variably primitive and pleomorphic tumor cells with scattered rhabdomyoblastic strap cells. External pathology review confirmed the diagnosis of embryonal rhabdomyosarcoma of the uterus.

She was referred to Gynecologic Oncology for further evaluation and underwent pelvic MRI. The patient was thoroughly counseled regarding available treatment options (e.g., surgery, chemotherapy, or radiation), including potential risks, benefits, and expected outcomes. Considering her frailty, comorbidities, symptom burden, and the limited expected benefit of aggressive therapy given the tumor’s extensive involvement and poor prognosis, she ultimately elected to forgo curative treatment in favor of a comfort-focused, palliative approach. She passed away in September 2024.

## Discussion

Only a few cases of uterine ERMS in postmenopausal patients have been reported in the literature to date [7]. The rarity of this presentation in this demographic often leads to diagnostic delays because clinicians may not initially consider ERMS in the differential diagnosis of uterine masses or vaginal bleeding [8]. In this patient, the nonspecific symptom of intermittent bleeding was not promptly evaluated due to the patient’s hesitance to seek medical attention and discontinuities in follow-up across multiple hospitals and emergency department encounters. This contributed to a late presentation marked by an advanced, bulky tumor causing urinary obstruction and life-threatening complications such as pulmonary embolism and sepsis.

Postmenopausal bleeding or spotting, even if intermittent or mild, warrants prompt and thorough evaluation. Early pelvic ultrasound is a widely available and valuable initial tool for evaluating postmenopausal bleeding and may facilitate earlier detection of underlying pathology, including solid and premalignant uterine lesions. While common etiologies include benign atrophic changes or endometrial hyperplasia, rare but aggressive malignancies such as ERMS should not be overlooked, particularly when bleeding persists or worsens [9].

A systematic review of uterine ERMS in 116 adult femal patients reported a mean age at diagnosis of 36.6 years, with vaginal bleeding as the most common presenting symptom and a median tumor size of 5 cm (range 0.5-27 cm) [10]. Our patient also first presented with bleeding at 76 years of age, well beyond the typical reported age range. Her tumor measured 13.5 cm, larger than the median reported in the literature, and was discovered only after bleeding progressed and systemic complications developed. Speculum examination at the time of our gynecologic evaluation revealed a visible tumor protruding from the vaginal canal and extending along the sidewalls and cervix. Although imaging demonstrated endometrial thickening and a large uterine mass suspicious for malignancy, earlier comprehensive pelvic examination, including speculum and bimanual assessment, may have allowed detection of gross vaginal and cervical involvement sooner, potentially expediting diagnostic evaluation. 

CT and MRI are useful for evaluating tumor size, local invasion, and extent of disease, while biopsy and immunohistochemistry remain essential for definitive diagnosis of ERMS [2]. In our case, the initial biopsy yielded only necrotic tissue, a diagnostic pitfall frequently encountered in high-grade sarcomas [11]. Repeat sampling revealed a poorly differentiated sarcomatoid malignancy characterized by spindled tumor cells, perivascular whorling, occasional “monster nuclei,” and a high mitotic index. Histologically, these aggressive features suggest a more virulent tumor phenotype in adults compared to children, likely contributing to rapid progression and poor prognosis [12].

In our patient, immunohistochemistry demonstrated focal positivity for desmin, myogenin, MyoD1, CK8/18, and CD10, as well as weak positivity for CKAE1/AE3 and p40. Markers for smooth muscle tumors (SMA), lymphoma (CD45), peripheral nerve sheath tumors (S100), vascular tumors (CD31, CD34), and PEComa (Melan-A) were negative. This profile excluded several mimics and supported a diagnosis of high-grade sarcoma with myogenic differentiation [13]. Adult uterine ERMS typically shows positivity for desmin, myogenin, and MyoD1, which are key markers of skeletal muscle differentiation and were expressed in 95.6% of tumors in a case series of 25 adult women [8]. The focal nature of skeletal muscle marker expression is consistent with adult uterine ERMS but provides weaker support than diffuse staining. Together with weak epithelial marker positivity (CKAE1/AE3 and p40), this unusual profile introduces diagnostic complexity and raises the possibility of mixed lineage differentiation. Staining patterns can be variable, particularly in necrotic or poorly differentiated tumors [14], as reflected in our patient’s findings. This emphasizes that ERMS may not always exhibit the classic immunohistochemical signature and suggests that molecular testing can be valuable when standard markers are inconclusive [2].

The aggressive biology of adult uterine ERMS, combined with the high prevalence of comorbidities in this population, complicates management. The pulmonary emboli that occurred months after the onset of vaginal symptoms likely indicate malignancy-associated hypercoagulability, further worsening her clinical course. Current treatment protocols for rhabdomyosarcoma are primarily based on pediatric data; however, multimodal approaches combining surgery, chemotherapy (e.g., the VAC (vincristine, adriamycin, cyclophosphamide) regimen), and radiation have been shown to improve survival in adults [15]. In elderly patients, treatment options are often limited by comorbidities and decreased tolerance for aggressive regimens. In such cases, management may need to be adapted to prioritize palliative care, focusing on quality of life rather than curative intent.

## Conclusions

Uterine ERMS in postmenopausal women is an extremely rare and aggressive malignancy associated with a poor prognosis. Its nonspecific and rapid presentation can lead to delays in diagnosis, particularly in elderly patients with comorbidities, which may limit the practicality of aggressive interventions. Clinicians should maintain a high index of suspicion when evaluating abnormal gynecological symptoms and encourage patients to report changes promptly. When curative therapy is not feasible, early integration of palliative care is critical for symptom management, preserving quality of life, and supporting patient-centered decision-making. Awareness of rare malignancies such as ERMS can facilitate timely diagnosis and support risk stratification to inform goal-concordant care, improving outcomes for vulnerable populations.
